# Towards sustainable pharmaceutical QC: micellar-UPLC strategy for concomitant analysis of a cardiovascular polypill with aniline and salicylic acid impurities

**DOI:** 10.1038/s41598-025-27968-w

**Published:** 2026-01-22

**Authors:** Ahmed R. Mohamed, Israa M. Nour, Mohamed Badrawy, Eman Darweish

**Affiliations:** Pharmaceutical Chemistry Department, Faculty of Pharmacy, Egyptian Russian University, Badr City, 11829 Cairo Egypt

**Keywords:** Micellar UPLC, Cardiovascular polypill, Drug impurities, Whiteness and greenness., Chemistry, Drug discovery, Health care, Medical research

## Abstract

Medication compliance is a key factor in achieving positive health outcomes, and this is difficult for patients, especially the elderly, who need polytherapy. Therefore, pharmacy experts have devised a single-pill combination that combines more than one active pharmaceutical ingredient to treat multiple diseases and improve patient compliance with medication. Here, an affordable UPLC approach was designed for the estimation of a fixed-dose combination that cures hypertension, hyperlipidemia, and blood coagulation and subsequently prevents heart attacks. This combination includes four active ingredients: aspirin (ASR), atorvastatin calcium (ATC), metoprolol succinate (MES), and ramipril (RAP). The applied approach was devised to determine the mentioned drugs in their commercial dosage form and also in the presence of ASR’s impurity (salicylic acid) and ATC’s impurity (aniline). The target drugs were separated on a Kinetex^®^ XB-C18 column using an isocratic micellar eluent system composed of 0.02 M Brij-35 at pH 3.0 (using orthophosphoric acid) and 10% n-propanol, flowed at 0.20 mL/min. The mentioned drugs were UV-scanned at 230.0 nm. ICH requirements were followed for all validation items. Whiteness and greenness appraisals were performed, affirming the applied approach’s friendliness to the environment. The priority of this UPLC approach over the published LC ones is due to using a micellar moving phase, making it more eco-friendly to the environment, and the determination of the aforementioned drugs even in the presence of aniline (toxic impurity) and salicylic acid.

## Introduction

Cardiovascular diseases, along with hyperlipidemia, rank among the top causes of mortality across the globe today. Furthermore, hypertension is a primary contributor to cardiovascular diseases and premature death around the world. The global prevalence of hypertension is rising due to the aging population, and it is worsened by lifestyle-related risk factors like unhealthy eating habits and insufficient physical activity^1^. Nonetheless, many different combinations of medicines have substantially improved and increased hypertension treatment over the previous two decades^2,3^.

Elevated cholesterol can result in the accumulation of fatty deposits within your blood vessels. These deposits can hinder blood circulation and potentially lead to a sudden clot, which might result in a stroke or a heart attack. High blood pressure can also be one of these drawbacks^4^. Consequently, uncontrolled high blood pressure can considerably affect heart disease and other essential organs, such as your kidneys and brain. So, it’s crucial to keep an eye on and manage your cholesterol levels to safeguard your heart’s well-being. Following the latest findings from a big European trial, a combination of specific blood pressure-lowering medications with cholesterol-lowering drugs may decrease heart attacks and strokes by half in adults with moderate cardiovascular risk^5^.

ASR is a nonsteroidal anti-inflammatory medication that contains acetylated salicylate (acetylsalicylic acid). It alleviates symptoms associated with inflammation and has a broad spectrum of pharmacologic activity, such as antiplatelet, analgesic, and antipyretic^6^. ATC is utilized to lower LDL cholesterol and triglycerides by inhibiting HMG-CoA reductase^7^. RAP has the ability to inhibit angiotensin-converting enzyme (ACE), leading to preventing blood vessels from contracting^8^. MES, a β-blocker medication, is utilized to treat hypertension and angina, causing lower mortality rates accompanying myocardial infarction^9^.

**Fig. 1:**
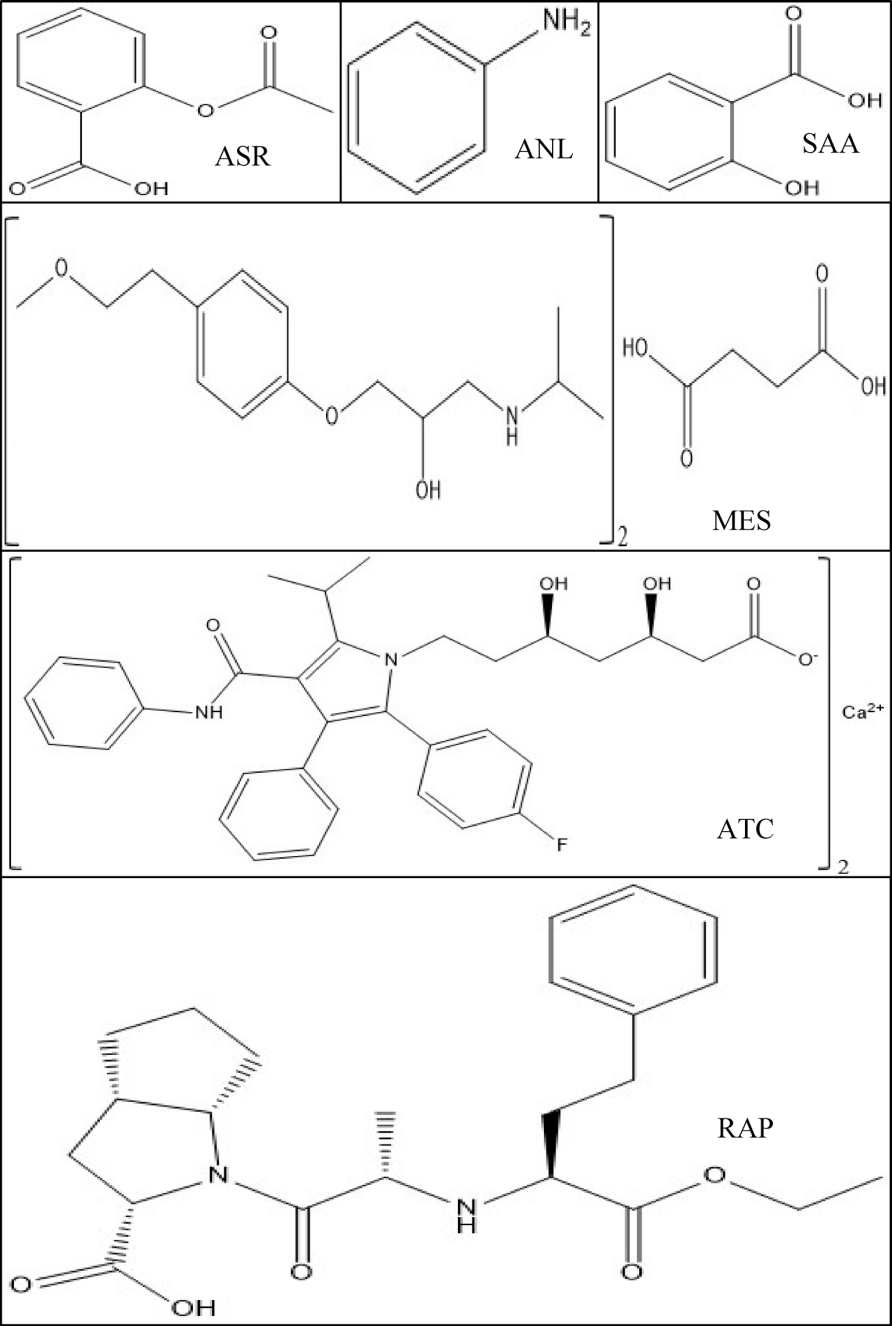
Chemical structures of the analytes under study.

Salicylic acid (SAA) has keratolytic properties; it is in a similar category of medications to ASR (salicylates). It works by boosting skin hydration and removing the constituent that empowers skin cells to remain stuck together. SAA is considered an impurity for ASR^10^. According to the USP–NF monograph for ASR tablets, the acceptance criterion for free SAA is not more than 3.0% of ASR’s labeled amount^11^. Aniline (ANL) is considered an organic compound that serves as a precursor to a variety of industrial compounds. ANL degrades hemoglobin, a protein in the blood that typically delivers oxygen. The damaged hemoglobin is unable to transport oxygen^12^. Additionally, exposure to ANL causes spleen and neuron poisoning, as well as other neurological consequences and sarcoma, which is characterized by splenomegaly, hyperplasia, fibrosis, and tumor growth towards the end^13^. ANL, according to this study^14^, is considered an impurity for ATC. The above-mentioned analytes’ chemical structures are presented in Fig. 1.

Separation and estimation of drug-related impurities is considered a critical step, as these impurities can have an impact on human life in addition to drug efficiency. As a result, specific regulations governing impurities have been developed by varied authorities of global regulatory bodies like the FDA, EMEA, and ICH. As it is critical to identify and separate any potential contaminants, monitoring them should be feasible^15,16^. So, the established procedures were modified to maintain environmental safety in addition to maintaining the efficacy of the proposed approaches. The chromatographic approaches were chosen because of their superior benefits in terms of small sample quantities, accurate, exact separation, and the ability to separate substances with identical chemical and physical characteristics^17–19^. UPLC is an efficient chromatographic technology that has a wide flow range and considerably decreases analytical time. The fundamental idea behind UPLC is that as the size of the fill particles reduces, so does the efficiency and hence the resolution^20^.

A review of the available literature revealed that only a few approaches have been reported for the quantification of the studied drugs (ASR, ATC, RAP, and MES)^21–23^, while no methodology has yet been established for their estimation in the presence of any of their related impurities. Therefore, this work aims to develop the first eco-friendly, sensitive, and efficient micellar-UPLC method, offering a green and rapid alternative for the simultaneous assay of this clinically important drug mixture in the presence of SAA and ANL (related impurities).

## Experimental

### Materials and reagents

All chemicals and solvents involved in this procedure are of HPLC grade, ensuring the highest standards of quality and reliability.

- ASR standard was sourced from Marcyrl Pharmaceuticals, Egypt (99.80%, certified purity).
- ATC and RAP standards were sourced from EIPICO, Egypt (99.80% and 99.70%, certified purities, in order).
- MES standard was sourced from AstraZeneca, Egypt (99.85%, certified purity).
- ANL was procured from Loba Chemie Pvt. Ltd., India (99.00%, certified purity).
- SAA was procured from Adwic, Egypt (99.20%, certified purity).
- Brij-35, n-propanol, and ethanol (Merck, Germany).

— Brij-35 (polyoxyethylene (23) lauryl ether), a non-ionic surfactant with the approximate formula C_12_H_25_(OC_2_H_4_)_23_OH, was employed as the micellar component of the eluent system. Brij-35 exhibits a high hydrophilic-lipophilic balance (HLB ≈ 16.9), excellent water solubility, and notable biodegradability^24^.

- Orthophosphoric acid (Fisher Scientific, UK).

### Pharmaceutical formulation

Zycad 4 MG^®^ Capsule; synthesized by Zydus Cadila, India; labeled to comprise 10 mg ATC, 75 mg ASR, 50 mg MES, and 5 mg RAP per capsule.

### Instruments

- The chromatographic setup consists of an Agilent 1100 series UPLC system (USA), which features a quaternary pump (genre G1311A), a variable-wavelength detector (genre G1314A), a degasser (genre G1322A), and a 1-µL sample loop automatic injector. Separations were accomplished on a Kinetex^®^ XB-C18 (100 Å, 1.70 μm, 2.10 × 50 mm) column.
- pH meter (genre Jenway 3510).
- Sonicator (genre WUC-Ao6H).

### Separation conditions

The optimal separation was performed using a Kinetex^®^ XB-C18 column at ambient temperature. Isocratic elution was achieved with a degassed and filtered solution comprising Brij-35 (0.02 M) at pH 3.0 (amended with orthophosphoric acid, which was added dropwise to the Brij-35 solution with continuous stirring until reaching this pH, as monitored using a pH meter) and 10% n-propanol. The flow rate of the aforesaid solution was set at 0.20 mL/min, while detection was carried out using an ultraviolet detector at a wavelength of 230.0 nm.

### Standard solutions

To create 5000 µg/mL stock solutions of ASR, ATC, MES, RAP, and SAA, 500 milligrams of each pure drug was sonicated in 90 mL ethanol within a 100 mL calibrated flask. Then, each flask volume was totaled to 100 mL using ethanol. During analysis, the aforesaid stock solutions (5000 µg/mL) were diluted, employing the moving phase to prime a 500 µg/mL working solution for each drug.

To create a 5100 µg/mL stock solution of ANL, 1 mL was conveyed from its standard liquid (1.02 gm/mL) into a 200 mL calibrated flask holding 199 mL ethanol. During analysis, the aforesaid stock solution (5100 µg/mL) was diluted, employing the moving phase to prime a 500 µg/mL working solution.

## Procedures

### Construction of calibration curves

Initially, the Kinetex column was equilibrated using the moving phase for 30 min before the compounds were injected. Precisely measured milliliters of ASR, ATC, SAA, MES, RAP, and ANL were conveyed from their respective 500 µg/mL working solutions, using suitable intermediate dilutions when necessary, into six separate series of six 10-mL calibrated flasks. Then, each flask volume was brought up to 10 mL with the moving phase to prime concentrated solutions varying from 5 to 90 µg/mL for ASR, 5–50 µg/mL for ATC, 5–60 µg/mL for MES, 2–50 µg/mL for RAP, 2–60 µg/mL for SAA, and 1–40 µg/mL for ANL. Following this, the solutions of each series were auto-injected (in 1-µL volumes) into the Kinetex column as described in the separation conditions section. Finally, a calibration graph was created for each compound: ASR, ATC, MES, RAP, SAA, and ANL, by plotting the areas beneath the peaks in relation to their corresponding concentrations.

### Application to pure drugs

Precisely measured milliliters were conveyed from the respective working solution (500 µg/mL) of each drug and impurities (SAA and ANL) into a series of five 10-mL calibrated flasks, then totaled to 10 milliliters with the moving phase. Hence, the flasks held five ratios of these compounds within their linearities. Subsequently, the primed solutions were auto-injected (in 1-µL volumes) into the Kinetex column as described in the separation conditions section. Lastly, the corresponding concentrations of each drug and impurities were estimated from their respective calibrations.

### Application to dosage form

After weighing twenty capsules with precision, the average mass of each capsule was determined, and the amount equivalent to the content of one capsule was weighed. Using the sonicator for 3 min, all active components were mined twice from inactive ingredients with 25 mL ethanol in a 100-mL flask. Afterward, the developing solution was carefully filtered into a new 100-mL flask, and its volume was totaled to 100 mL using ethanol. Ethanol was also utilized to efficiently cleanse the residues after filtration, ensuring optimal extraction, before filling the flask to its mark. Then, we diluted the aforesaid pharmaceutical solution into a 10-mL flask using the moving phase, getting each medication within its linearity. As emphasized in the separation conditions section, the final primed sample underwent auto-injection (into the Kinetex column) and chromatographic analysis in triplicate. Finally, the nominal contents of the four medications in their capsules, other than their crude concentrations (using the conventional addition method), were accurately determined using their corresponding calibrations.

## Results and discussion

This effort focuses on developing the first micellar UPLC platform for concurrently determining ATC, RAP, ASR, and MES in their polypill. This method, which relies on avoiding hazardous solvents that harm the environment, decisively outperforms existing LC techniques. With this innovative approach, we are setting a new standard in quality control that prioritizes both excellence and sustainability. Furthermore, the separation and analysis of drug-related impurities are viewed as an essential process since these impurities can affect human health as well as the effectiveness of the medication. Consequently, this platform was designed to estimate the aforementioned drugs, besides ASR and ATC impurities (SAA and ANL), using UPLC separation that demonstrated high resolution, minimal solvent usage, and quick analysis time.

### Method design and optimization

This research aims to optimize the chromatographic separation of target drugs and two of their impurities (SAA and ANL), minimizing analysis time and environmental impact. Hence, a series of experiments was performed to evaluate the influence of different columns, moving phase compositions, pH values, flow rates, and scanning wavelengths on separation efficiency.

### Column

Two types of columns were evaluated: Kinetex C8 and Kinetex C18. The C8 column’s insufficient resolution led to the sample components co-eluting, impeding accurate identification and quantitation. Conversely, the Kinetex C18 column provided benefits such as lower mobile phase usage, rapid (9.30-minute) elution with sufficient resolution, and excellent system suitability, making it a highly efficient choice for this analysis.

### Eluent system

Micellar moving phases were selected for this study due to their cost-effectiveness, high elution strength, and reduced environmental impact^25^. Hence, a moving system consisting of a micellar solution with a small volume of a green organic modifier was adopted in this study. To boost separation performance while minimizing ecological impact, the following variables were optimized: surfactant (Brij-35) concentration, organic modifier (n-propanol) percentage, and pH.

**Fig. 2:**
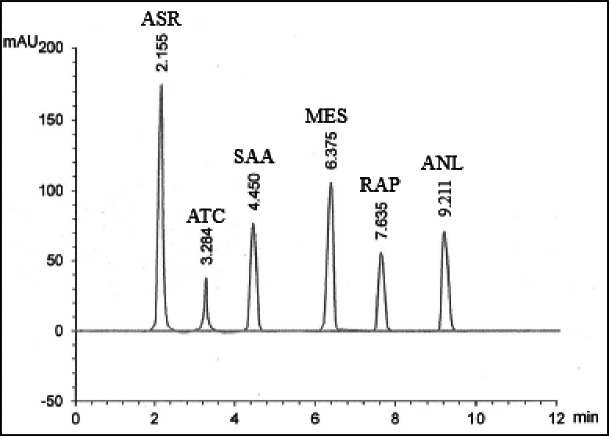
UPLC-chromatogram of ASR (t_R_ = 2.155 min), ATC (t_R_ = 3.284 min), SAA (t_R_ = 4.450 min), MES (t_R_ = 6.375 min), RAP (t_R_ = 7.635 min), and ANL (t_R_ = 9.211 min) under the specified chromatographic conditions.

In a series of experiments, we tested several combinations of n-propanol (5% to 15%) and Brij-35 (0.01 M to 0.04 M) with pH ranging from 2.5 to 5 (adjusted with orthophosphoric acid). We found that n-propanol percentages deviating from 10% led to either unacceptable peak delays (below 10%) or unresolved peaks (above 10%). Similarly, Brij-35 concentrations above 0.02 M resulted in peak asymmetry, while concentrations below 0.02 M increased retention times (t_R_) and broadened peaks. For pH, deviations from 3 resulted in either excessively rapid elution (below 3) or unacceptably delayed peaks (above 3). So, 0.02 M Brij-35 at pH 3.0 with 10% n-propanol yielded optimal results, achieving a 9.30-minute elution time (Fig. 2).

### Flow rate

Efficient separation of the analyzed peaks (drugs and impurities) within a reasonable timeframe was achieved using a 0.20 mL/min flow rate. This was determined through an evaluation of flow rates, which varied between 0.10 and 0.50 mL/min, and their influence on retention times.

### Detection wavelength

In UV detection trials across all tested compounds, a wavelength of 230.0 nm proved to be the most efficient. This setting provided the strongest signal and consistently yielded sharp, well-defined peaks.

Ultimately, optimal separation was achieved using a Kinetex^®^ XB-C18 column at ambient temperature, employing isocratic elution with a degassed and filtered solution of 0.02 M Brij-35 (pH 3.0, amended with orthophosphoric acid) and 10% n-propanol at 0.20 mL/min, with UV detection at 230.0 nm. Under these conditions, the t_R_ values were 2.155, 3.284, 4.450, 6.375, 7.635, and 9.211 min for ASR, ATC, SAA, MES, RAP, and ANL, in order (Fig. 2).

### Method validation

The UPLC technique recommended in this study was validated pursuant to ICH recommendations^26^ in the following manner:

### Linearity

By calculating the integrated peak areas from different concentrations of each ASR, ATC, MES, RAP, SAA, and ANL, the linearity of the suggested platform was assessed under ideal chromatographic settings. Plotting the concentrations of each compound in µg/mL against their corresponding peak areas (at 230.0 nm) allowed calibration graphs for the examined compounds to be produced. The suggested approach demonstrated adequate linearity within the ranges of 5–90 µg/mL for ASR, 5–50 µg/mL for ATC, 5–60 µg/mL for MES, 2–50 µg/mL for RAP, 2–60 µg/mL for SAA, and 1–40 µg/mL for ANL. Excellent linearity was observed over the studied concentration ranges, as indicated by correlation coefficients (r) exceeding 0.9996 for all analytes. Subsequently, each compound’s regression equation and important analytical parameters were determined and shown in Table 1.

**Table 1:** Assay parameters for the determination of the studied analytes by the suggested method.

Analytes Parameters	ASR	ATC	MES	RAP	SAA		ANL
Linearity range (µg/mL)	5–90	5–50	5–60	2–50	2–60	1–40	
Correlation coefficient (r)	0.9998	0.9997	0.9998	0.9999	0.9999	0.9999	
Slope	15.11	13.10	10.52	10.03	12.12		11.22
Intercept	−35.50	−26.73	−32.85	−4.02	−4.19		4.11
S.D of intercept ^a^	6.10	4.53	3.34	1.18	2.16		1.04
LOD (µg/mL)	1.33	1.14	1.05	0.39	0.59		0.31
LOQ (µg/mL)	4.04	3.46	3.17	1.18	1.78		0.93
Accuracy (Mean ± SD) ^b^	99.16 ± 1.23	101.03 ± 1.11	98.15 ± 1.34	99.85 ± 1.05	99.20 ± 0.95		100.34 ± 1.07
Intra-day precision (RSD%) ^c^	0.84	1.12	1.26	0.97	1.08		0.73
Inter-day precision (RSD%) ^d^	1.03	1.19	1.45	1.20	1.16		0.97

^a^ Standard deviation of intercept.

^b^ Mean of five determinations.

^c^ Intra-day precision; relative standard deviation of (10, 40, and 80 µg/mL) of ASR, (5, 20, and 35 µg/mL) of ANL, and (10, 25, and 45 µg/mL) of ATC, MES, RAP, and SAA in triplicate within the day.

^d^ Inter-day precision; relative standard deviation of (10, 40, and 80 µg/mL) of ASR, (5, 20, and 35 µg/mL) of ANL, and (10, 25, and 45 µg/mL) of ATC, MES, RAP, and SAA in triplicate on three consecutive days.

### LOD and LOQ

To evaluate the sensitivity of the proposed method, the detection (LOD) and quantitation (LOQ) parameters were estimated using the slope (S) and the standard deviation of the intercept of the calibration plot (σ) for every analyte, as shown:

$${\rm LOD =(3.3 x \sigma)/S\:\:\:\:\:\:\:\:\:\:\:\:\:\:\:\:\:\:\:\:LOQ =(10 x \sigma)/ S}.$$

The findings listed in Table 1 demonstrate the excellent sensitivity of the proposed platform for analyzing the target compounds.

### Accuracy and precision

To ensure accurate and precise measurements at both intra- and inter-day levels, three distinct pure concentrations of each drug were carefully generated to cover the low, medium, and high ranges of their linearity. The concentrations that were prepared were then tested three times using our method. All findings are listed in Table 1, confirming our method’s excellence in accuracy and precision.

### Specificity

**Fig. 3:**
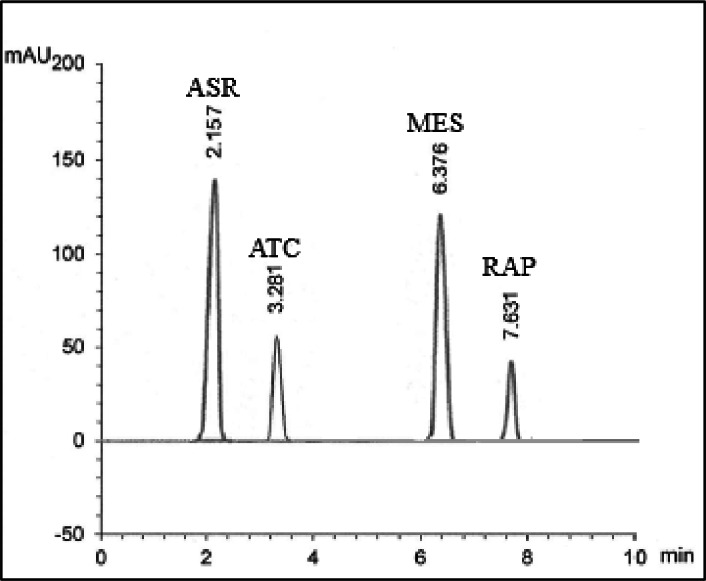
UPLC-chromatogram of ASR (75 µg/mL), ATC (10 µg/mL), MES (50 µg/mL), and RAP (5 µg/mL) in Zycad 4 MG^®^ Capsule.

Specificity was confirmed by successfully separating and quantifying the studied compounds in laboratory mixtures (Fig. 2; Table 2) and in pharmaceutical capsules, demonstrating a lack of interference from excipients (Fig. 3; Table 3).

**Table 2:** Determination of the studied analytes in their laboratory-made mixtures by the suggested method.

Mix	ASR		ATC		MES		RAP		SAA		ANL	
	Added^a^	*R*%^b^	Added^a^	*R*%^b^	Added^a^	*R*%^b^	Added^a^	*R*%^b^	Added^a^	*R*%^b^	Added^a^	*R*%^b^
Mix 1	60	99.14	40	98.59	40	100.77	40	101.22	15	98.99	10	99.02
Mix 2	30	98.67	30	98.37	30	99.59	30	101.64	20	99.60	10	98.90
Mix 3	50	100.12	25	100.34	50	101.03	25	100.07	50	98.79	25	99.44
Mix 4	15	99.07	30	99.92	15	99.40	30	99.73	35	100.04	20	101.06
Mix 5^c^	75	100.56	10	99.56	50	100.40	5	100.66	25	99.88	30	100.91
Mean ± SD	99.51 ± 0.79		99.36 ± 0.85		100.24 ± 0.72		100.66 ± 0.79		99.46 ± 0.55		99.87 ± 1.04	

^a^ Added (µg/mL).

^b^ Recovery% (mean of three determinations).

^c^ The same concentration ratio in Zycad 4 MG^®^ Capsule.

**Table 3:** Assay of the four drugs in their capsules and application of standard addition approach.

Product content		Product (Found% ± SD) ^b^	Standard addition (Pure found% ± SD) ^b^
Zycad 4 MG^®^ Capsule ^a^	ASR	99.32 ± 1.04	98.88 ± 1.21
	ATC	98.78 ± 1.15	99.87 ± 1.29
	MES	99.54 ± 0.89	100.39 ± 0.97
	RAP	99.05 ± 1.33	98.89 ± 1.39

^a^ Zycad 4 MG^®^ Capsule; labeled to comprise 10 mg ATC, 75 mg ASR, 50 mg MES, and 5 mg RAP per capsule.

^b^ Mean of five determinations.

### Robustness

By replicating the previously specified chromatographic conditions and marginally adjusting specific experimental parameters, including scanning wavelength (± 2.0 nm), flow rate (± 0.02 mL/min), moving phase ratio (n-propanol, ± 2.0%), and pH (± 0.10), the robustness of our method was appraised. It was observed that the suggested method exhibited consistent performance with no discernible differences under the varied conditions (Table 4), thus affirming the method’s stability and reliability.

**Table 4:** Robustness study of the suggested method using concentration (30 µg/mL) for each studied analyte.

Variation		ASR	ATC	MES	RAP	SAA	ANL
		Recovery% ^a^ ± SD					
No variation (optimal conditions ^b^)		99.52 ± 0.98	99.87 ± 0.82	100.65 ± 1.08	100.33 ± 1.10	101.01 ± 1.07	99.94 ± 0.96
Moving phase (n-propanol ratio)	12%	99.14 ± 0.64	99.56 ± 0.89	98.67 ± 0.94	99.58 ± 1.36	99.74 ± 0.97	98.98 ± 1.29
	8%	99.16 ± 1.07	98.85 ± 0.99	98.79 ± 1.47	99.50 ± 1.28	99.19 ± 1.03	100.57 ± 1.43
Flow rate	0.22 mL/min	100.78 ± 1.21	101.63 ± 1.52	100.89 ± 1.57	100.97 ± 1.30	99.06 ± 1.26	99.75 ± 1.22
	0.18 mL/min	100.52 ± 1.17	100.09 ± 1.27	100.95 ± 1.38	98.51 ± 0.90	99.60 ± 1.49	99.07 ± 1.59
pH	3.10	99.78 ± 0.78	98.39 ± 1.10	98.94 ± 1.02	99.18 ± 0.87	99.48 ± 1.63	99.05 ± 1.40
	2.90	100.52 ± 1.60	101.74 ± 1.53	101.07 ± 1.27	99.39 ± 1.40	98.89 ± 1.47	101.78 ± 1.21
Scanning wavelength	232 nm	99.32 ± 1.01	101.04 ± 1.12	100.30 ± 1.02	99.96 ± 0.99	99.08 ± 1.07	99.78 ± 1.16
	228 nm	98.82 ± 1.31	98.93 ± 1.04	99.55 ± 1.17	100.53 ± 1.39	100.28 ± 1.01	99.06 ± 1.20

^a^ Mean of three determinations.

^b^ Optimal conditions: moving phase composition (0.02 M Brij-35 at pH 3.0 with 10% n-propanol), flow rate (0.20 mL/min), and scanning wavelength (230 nm).

### System suitability

To ensure the proper functioning of the eluent system, a thorough examination of the system suitability parameters was conducted. These parameters included resolution, tailing factor, capacity factor, selectivity factor, and theoretical plates’ number (a measure of column competence). The calculated parameters, as presented in Table 5, were all within the established acceptable limits^27^. This finding demonstrates that the proposed method is appropriate for the separation and quantification of the four drugs in addition to their impurities (SAA and ANL) under optimal conditions.

**Table 5:** System suitability parameters of the suggested method for the determination of the studied analytes.

Parameters	ASR	ATC	SAA	MES	RAP	ANL	Reference value^27^
Retention time (t_R_, min)	2.155	3.284	4.450	6.375	7.635	9.211	–
Resolution (Rs)	8.39	8.27	9.03	8.32	8.91		> 2
Capacity factor (K′)	2.54	2.89	3.21	3.65	2.82	2.66	1 < K′<10
Selectivity factor (α)	8.51	8.82	8.06	8.27	7.38		> 1
Tailing factor (T)	1.07	1.08	1.04	1.02	1.09	1.13	< 2
Theoretical plates’ number (N)	5543	6398	5471	5969	6605	7477	> 2000

### Application to dosage form

The suggested UPLC platform successfully quantified ASR, ATC, RAP, and MES in Zycad 4 MG^®^ capsules without interference from pharmaceutical additives. Satisfactory tabulated data, including standard addition recovery values, support the UPLC platform’s adoption for routine QC analysis (Table 3).

## Statistical comparison

Statistical analysis was conducted to compare the experimental outcomes from the designated UPLC platform with those from the documented HPLC^22^ using t- and F-tests. Based on the t- and F-values shown in Table 6, there is no statistically notable divergence between the two techniques, thereby affirming the equivalent precision and accuracy of our platform.

**Table 6:** Statistical comparison between the suggested and reported^22^ methods for the analysis of the four drugs in their pure forms.

Methods Parameters	Suggested method				Reported method^22^			
	ASR	ATC	MES	RAP	ASR	ATC	MES	RAP
Mean	100.56	98.93	99.86	100.17	100.24	99.14	99.63	99.49
SD	1.09	1.02	1.14	1.11	1.05	0.95	1.20	0.86
N	5	5	5	5	5	5	5	5
Variance	1.19	1.04	1.30	1.23	1.10	0.90	1.44	0.74
Student^’^s *t*-test (2.31)*	0.47	0.34	0.31	1.08	-------	-------	-------	-------
F- value (6.39)*	1.08	0.87	0.90	1.67	-------	-------	-------	-------

*The parentheses contain the corresponding theoretical *t* and F values at (*P* = 0.05).

## Whiteness and greenness appraisals

**Fig. 4:**
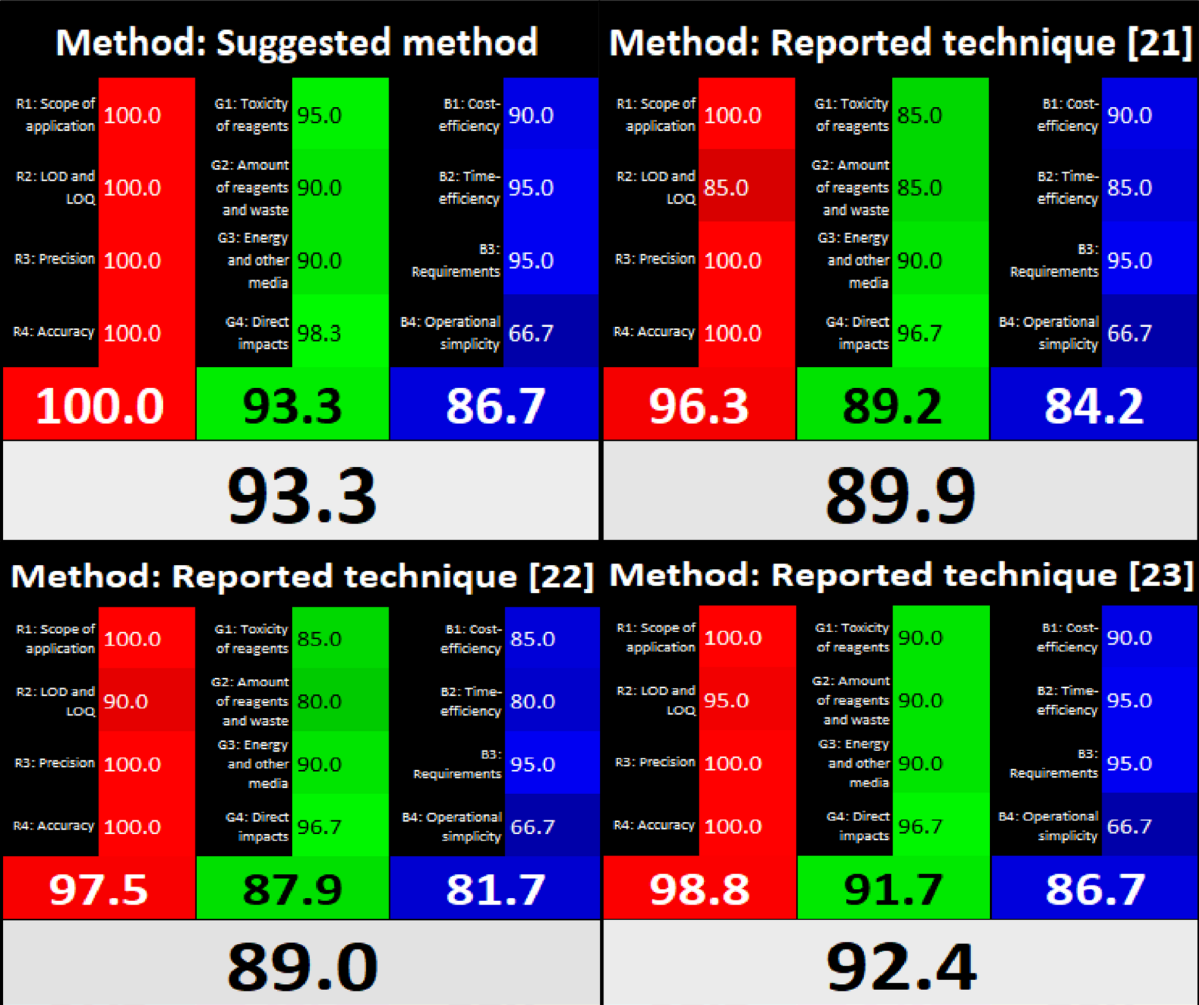
Whiteness evaluation of the suggested and reported^21–23^ methods by the RGB-colored model.

The “whiteness” and “greenness” of our technique and the documented ones^21–23^ were quantitatively evaluated using the RGB-colored model^25,28,29^ and the eco-scale metric^25,29,30^, respectively. The RGB-colored model, which uses twelve Excel-driven algorithms organized into blue, green, and red categories to evaluate the sustainability of procedures, showed our technique’s superior “whiteness” (Fig. 4). In parallel, the eco-scale metric, a system assigning penalty points for aspects impacting the analytical process, revealed fewer penalty points awarded to our platform (Table 7) as compared to the documented methods, thus affirming its superior greenness.

**Table 7:** Results of eco-scale analysis for the suggested and reported^21–23^ methods.

Methods Parameters	Suggested method	Reported method^21^	Reported method^22^	Reported method^23^
**Reagents**				
Methanol	–	12	12	–
Ethanol	4	–	–	–
Acetonitrile	–	–	8	8
n-Propanol	0	–	–	–
Brij-35	0	–	–	–
Orthophosphoric acid	2	–	2	–
Dipotassium hydrogen phosphate	–	–	0	0
Disodium hydrogen phosphate	–	–	–	0
Glacial acetic acid	–	–	–	2
Toluene	–	6	–	–
Ethyl acetate	–	4	–	–
Formic acid	–	6	–	–
**Instruments**				
Energy	1 [> 0.1 kWh/sample]	1 [> 0.1 kWh/sample]	1 [> 0.1 kWh/sample]	1 [> 0.1 kWh/sample]
Occupational hazard	3	3	3	3
Waste	5	5	5	5
Total penalty points	Σ 15	Σ 37	Σ 31	Σ 19
Analytical eco-scale total score ^a, b^	85	63	69	81
	Excellent green analysis	Acceptable green analysis	Acceptable green analysis	Excellent green analysis

^a^ Analytical eco-scale total score = 100–total penalty points.

^b^ If the score is > 75, it indicates excellent green analysis.

If the score is > 50, it indicates acceptable green analysis.

If the score is < 50, it indicates inadequate green analysis.

This superiority is primarily attributed to the use of a micellar eluent system containing only 10% n-propanol and the rapid separation of six analytes in just 9.30 min, which together resulted in the lowest penalty points and superior greenness compared to the documented methods^21–23^ that relied on toxic organic solvents to separate only four analytes over longer run times. The micellar component (Brij-35), with its biodegradable and non-toxic features, renders it a green solvent alternative. Its incorporation not only minimized the use of organic solvents but also contributed significantly to the method’s superior greenness and whiteness scores.

## Conclusion

For the first time, a sensitive and rapid micellar UPLC approach was designed and optimized to separate and quantify ASR, ATC, MES, and RAP in their capsules without excipients’ intrusion. Also, the method effectively resolved these drugs even in the presence of SAA and ANL impurities. Our UPLC platform offers significant advantages over the documented HPLC method, including a green micellar moving phase promoting environmental sustainability, a rapid 9.30-minute analysis time, and small LOD values, enabling affordable routine determination of the studied analytes in QC units. Assessment via the RGB-colored model and eco-scale metric affirms our method’s superior whiteness and greenness compared to the documented methods, establishing it as a superior alternative.

## Data Availability

All data generated or analyzed during this study are included in this published article.
